# Complete Biallelic Insulation at the *H19/Igf2* Imprinting Control Region Position Results in Fetal Growth Retardation and Perinatal Lethality

**DOI:** 10.1371/journal.pone.0012630

**Published:** 2010-09-08

**Authors:** Dong-Hoon Lee, Purnima Singh, Walter M. K. Tsark, Piroska E. Szabó

**Affiliations:** 1 Department of Molecular and Cellular Biology, City of Hope National Medical Center, Duarte, California, United States of America; 2 Transgenic Mouse Facility, City of Hope National Medical Center, Duarte, California, United States of America; Ludwig-Maximilians-Universität München, Germany

## Abstract

**Background:**

The *H19*/*Igf2* imprinting control region (ICR) functions as an insulator exclusively in the unmethylated maternal allele, where enhancer-blocking by CTCF protein prevents the interaction between the *Igf2* promoter and the distant enhancers. DNA methylation inhibits CTCF binding in the paternal ICR allele. Two copies of the chicken β-globin insulator (ChβGI)_2_ are capable of substituting for the enhancer blocking function of the ICR. Insulation, however, now also occurs upon paternal inheritance, because unlike the *H19* ICR, the (ChβGI)_2_ does not become methylated in fetal male germ cells. The (ChβGI)_2_ is a composite insulator, exhibiting enhancer blocking by CTCF and chromatin barrier functions by USF1 and VEZF1. We asked the question whether these barrier proteins protected the (ChβGI)_2_ sequences from methylation in the male germ line.

**Methodology/Principal Findings:**

We genetically dissected the ChβGI in the mouse by deleting the binding sites USF1 and VEZF1. The methylation of the mutant versus normal (ChβGI)_2_ significantly increased from 11% to 32% in perinatal male germ cells, suggesting that the barrier proteins did have a role in protecting the (ChβGI)_2_ from methylation in the male germ line. Contrary to the *H19* ICR, however, the mutant (mChβGI)_2_ lacked the potential to attain full de novo methylation in the germ line and to maintain methylation in the paternal allele in the soma, where it consequently functioned as a biallelic insulator. Unexpectedly, a stricter enhancer blocking was achieved by CTCF alone than by a combination of the CTCF, USF1 and VEZF1 sites, illustrated by undetectable *Igf2* expression upon paternal transmission.

**Conclusions/Significance:**

In this in vivo model, hypomethylation at the ICR position together with fetal growth retardation mimicked the human Silver-Russell syndrome. Importantly, late fetal/perinatal death occurred arguing that strict biallelic insulation at the *H19/Igf2* ICR position is not tolerated in development.

## Introduction

Enhancers are capable of activating gene promoters from great distances. It is the role of insulators in the genome to inhibit promiscuous long range activation of promoters [1], [2], [3]. Insulator action can manifest in enhancer blocking and chromatin barrier functions [2], [4]. Enhancer blocking means that an insulator is located between enhancer and promoter elements and prevents their communication. Chromatin barriers function to demarcate active and repressive chromatin domains. CCCTC binding factor (CTCF) [5], [6], [7] is the major insulator protein in vertebrates [5]. The enhancer-blocking role of the CTCF protein has been confirmed in various in vitro and in vivo transgenic assays and in genetic studies in the mouse [8], [9]. In the context of the genome, in vivo CTCF binding is often associated with sharp chromatin transitions, indicative of the presence of chromatin barriers [10], [11]. CTCF, however, does not have barrier function [12]. Chromatin barrier function has recently been attributed to upstream stimulatory factor 1 (USF1) [13] and to vascular endothelial zinc finger 1 (VEZF1), also called beta globin protein 1 (BGP1) [14], [15], [16], [17], [18], [19], [20] in transgenic mouse experiments [21], [22].

**Figure 1:**
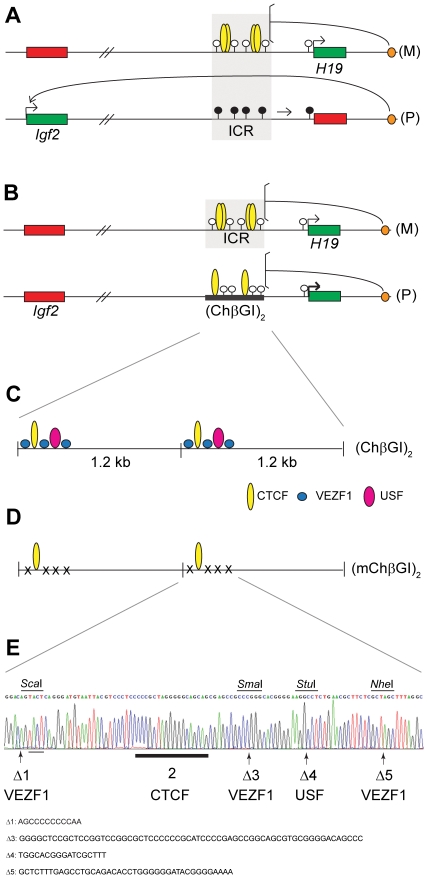
Imprinted versus non-imprinted insulation at the *H19/Igf2* locus by two distinct insulators. (A) Imprinted insulation at the *H19/Igf2* imprinted domain by the ICR. Maternal chromosome (M): unmethylated (white lollipops) ICR (shaded area) is inherited from the egg. CTCF (yellow ovals) imparts insulator activity (bracket) between the *Igf2* promoters and the shared, downstream enhancers (orange oval). Initiation of *H19* expression depends on an unmethylated ICR during embryogenesis. Paternal chromosome (P): methylated (black lollipops) ICR is inherited from the sperm, CTCF cannot bind, hence ICR has no insulator activity, *Igf2* promoters and enhancers can interact. Early in postimplantation development, the *H19* promoter is inactivated by an ICR-dependent mechanism (horizontal arrow). (B) Non-imprinted insulation at the *H19*/*Igf2* locus by the chicken β-globin insulator duplex (ChβGI)_2_ [44]. The (ChβGI)_2_ is unmethylated and insulates the *Igf2* promoter from the shared enhancers when substituted for the ICR and transmitted maternally (not shown) or paternally (P), with 10% *Igf2* activity remaining. *H19* is overactivated 1.5-fold by the (ChβGI)_2_ sequences in the paternal allele (bold arrow). (C) Structure of the (ChβGI)_2_ with the five in vitro footprints of the core insulator [8]: binding sites 1, 3 and 5 (blue circle): VEZF1 (BGP1); binding site 2: CTCF; and binding site 4 (pink oval): USF1. (D) Structure of the mutant chicken β-globin insulator duplex (mChβGI)_2_. Only the CTCF binding site (thick underlining) remains in each unit after deleting (x) binding sites 1, 3, 4 and 5 using site-directed mutagenesis. (E) Confirmation of the site-directed mutagenesis by DNA sequencing. Arrows indicate the positions of the deleted binding sites (deleted sequences shown underneath) and light underlining shows added nucleotides at footprint 1. Novel restriction sites, *Sca*I, *Stu*I and *Nhe*I, marked above, were generated to aid the screening of mutant colonies. One out of two *Sma*I sites remained at the footprint 3 deletion.

The chicken β-globin insulator (ChβGI) and the *H19/Igf2* imprinting control region (ICR) are two well-studied insulator regions. Both regions exhibit very strong insulation between an enhancer and promoter elements and their insulator function depends on CTCF binding. There is, however, a major difference between these two insulators in that the insulator activity of the *H19/Igf2* ICR depends on parental origin [23], [24], [25], [26]. The 2.4 kb long ICR [27], [28], [29], [30] is methylated in the sperm, but is unmethylated in the egg. This primary methylation difference (genomic imprint) is passed into the zygote, maintained during embryogenesis and determines the activity status of the ICR in the soma. The ICR is responsible for maternal allele specific expression of *H19* and for paternal allele specific expression of *Igf2* [28]. In the soma the maternally inherited unmethylated allele binds CTCF at four sites in vivo [26], [31], [32], [33], [34], resulting in insulation [34], [35], [36], [37], [38], [39] between the insulin-like growth factor 2 (*Igf2*) promoters and the shared downstream enhancers [40]. In contrast, in the paternally inherited ICR allele DNA methylation inhibits CTCF binding/enhancer blocking function, hence *Igf2* is expressed (Figure 1A). The paternally inherited ICR is also required for inactivating *H19* during early embryo development by methylation spreading [41]. Inactivation of the CTCF binding sites in the maternal allele results in the loss of enhancer-blocking activity in the maternal allele, biallelic *Igf2* expression and large fetus size [34], [35], [36], [37], [38], [39]. CTCF binding in the maternally inherited ICR is also required in the early embryo for initiation of *H19* expression [35], and for maintaining hypomethylation of the ICR in the soma [34], [35], [36], [37], [38], [39]. CTCF binding, however, is not responsible for the germ line events that establish the methylation differences at the ICR between egg and sperm. The CTCF site-mutant ICR was correctly unmethylated in female fetal germ cells [39] and ovulated oocytes [35], [38], [39], and it was correctly methylated in fetal male germ cells [39] and in sperm [35].

The 1.2 kb long ChβGI is located in the constitutive DNaseI hypersensitive site 4 between a 12 kb heterochromatin stretch and the β-globin locus in the chicken. In transgenic mice two copies of the 1.2 kb can protect transgenes from position effects [42], [43]. Most of the insulator activity resides in a 250 bp “core element” which contains five in vitro footprints (Figure 1C) [8]. Insulator function has been attributed to footprint 2 (CTCF) whereas chromatin barrier activity was associated with footprints 1, 3, 5 (BGP1/VEZF1) and 4 (USF) [12]. The barrier protein, USF is required for maintaining euchromatin features including histone 3 lysine 4 dimethylation (H3K4me2) and histone hyperacetylation of the ChβGI and the ChβGI-surrounded transgene sequences [22]. BGP1 (VEZF1) is important for maintaining euchromatin at the insulated transgene [22] and for maintaining DNA hypomethylation at the ChβGI and along the ChβGI-surrounded transgene [21].

We previously substituted the ICR with two copies of the chicken β-globin insulator, (ChβGI)_2_ [44]. The (ChβGI)_2_ lacks homology to the ICR except for the two CTCF sites. We found that due to CTCF binding, upon maternal transmission the (ChβGI)_2_ sequences substituted for the insulator function of the ICR: in fetal organs *Igf2* expression was very low in the mutant maternal allele. Upon paternal transmission, however, the (ChβGI)_2_, failed to undergo de novo methylation in the male germ line and remained unmethylated in the soma, resulting in biallelic insulation (Figure 1B). *Igf2* expression was reduced to 10% and fetus size was 50–61% of normal siblings. *H19* expression was biallelic and the paternal allele's expression was overactivated, it accounted for 77% of total *H19* expression in fetal livers and kidneys [44].

We now asked the question whether the regulatory elements that provide the (ChβGI)_2_ with barrier activity are responsible for the non-imprinted behavior of the (ChβGI)_2_ at the *H19* ICR position. We hypothesized specifically that, due to their euchromatin-maintaining potential, the USF and VEZF1 sites may protect the (ChβGI)_2_ from de novo methylation in the male germ line. This could occur if the genomic locus outside the *H19/Igf2* ICR carried clues for directing de novo methylation to the endogenous ICR or to an introduced DNA fragment, such as (ChβGI)_2_ at the ICR position. We tested this hypothesis by deleting the USF and VEZF1 binding sites from the (ChβGI)_2_ and used two copies of this mutant chicken β-globin insulator (mChβGI)_2_ to substitute for the *H19*/*Igf2* ICR. In this way enhancer blocking activity was maintained at the ICR position due to two intact CTCF sites but barrier activity was abolished because six VEZF1 and two USF sites were absent. We expected that when these barrier proteins were removed, the (mChβGI)_2_ could become methylated in the male germ line. If this methylation is maintained in the paternal allele in the soma, it would result in parental-allele specific *H19* and *Igf2* expression.

We found that the (mChβGI)_2_ attained significantly more methylation in fetal male germ cells than the normal (ChβGI)_2_, suggesting that the boundary proteins provided protection from methylation in the male germ line. This methylation, however was not maintained in the paternal allele, indicating that the (mChβGI)_2_ lacked the capacity for methylation maintenance in the soma. Therefore, similarly to the (ChβGI)_2_, the (mChβGI)_2_ was a biallelic insulator. Its paternal transmission resulted in biallelic *H19* expression and undetectable *Igf2* expression. The enhancer blocking function was, unexpectedly, stronger by CTCF alone than by using a combination of CTCF, USF and VEZF1 sites. Our results argue that complete biallelic enhancer blocking at the *H19/Igf2* ICR position results in perinatal lethality.

## Materials and Methods

The experiments involving mice had been approved by the IACUC of the City of Hope. Housing and care of the animals has been consistent with the Public Health Service Policy, the NIH “Guide for the Care and Use of Laboratory Animals” and the Animal Welfare Act.

### Site-directed mutagenesis

The mutator plasmid, pGEM4Z-link3, was generated by ligating annealed Link 3 polylinker oligos (5′-AATTCGAGCTCGGTACCGTCGACGCATGCTAGATCACGCGTA-3′ and 5′-AGCTTACGCGTGATCTAGCATGCGTCGACGGTACCGAGCTCG-3′) into *Hind*III and *Eco*RI double-digested pGEM4Z. The ChβGI fragment from plasmid pJC13-1 [9] was subcloned into the *Acc65*I site of pGEM4Z-link3. FP3 was deleted by *Sma*I digestion and religation. This plasmid was used for further mutagenesis using the Transformer Site-directed mutagenesis kit (BD Biosciences). Two selection primers were designed for plasmid pGEMZ such way that subsequent mutatagenesis cycles switch back-and forth between *Mlu*I and *Age*I sites. The *Age*I/*Mlu*I selection primer was 5′-TGCTAGATCACCGGTAAGCTTGTCTCCC-3′, containing an *Mlu*I site and the *Mlu*I/*Age*I selection primer was 5′- TGCTAGATCACGCGTAAGCTTGTCTCCC-3′, containing an *Age*I site. In the first site-directed mutagenesis cycle FP4 and FP5 were deleted (Figure 1) by the 5′- GCACGGGGAAGGCCTCTGAACGCT-3′ oligo containing a *Stu*I and the 5′-TCTGAACGCTTCTCGCTAGCTTTAGGCTGAA-3′ oligo containing a *Nhe*I site, respectively. In the second site-directed mutagenesis cycle, FP1 was mutated by deleting AGCCCCCCCCCAA and inserting TACT using the 5′- CTAGAGGGACAGTACTCAGGGATGTAATT-3′ oligo containing a *Sca*I site. The mutant clones were identified by restriction digestion and verified by DNA sequence analysis.

Two copies of the mChβGI were inserted into the *Sac* I and the *Eco*RI – *Sph*I positions of the acceptor plasmid, pGEM4Z-Link2. The acceptor plasmid was generated by ligating the annealed Link2 oligos (5′-AATTGGATCCGAGCTCGTCGACGAATTCGCATGCGGATCCA-3′ and 5′-AGCTTGGATCCGCATGCGAATTCGTCGACGAGCTCGGATCC-3′) into *Hind*III and *Eco*RI double digested pGEM4Z. The orientation of the inserts was verified by *Sca*I analytical digestion.

### Gene targeting to produce mouse lines with the ICR substitution

**Figure 2:**
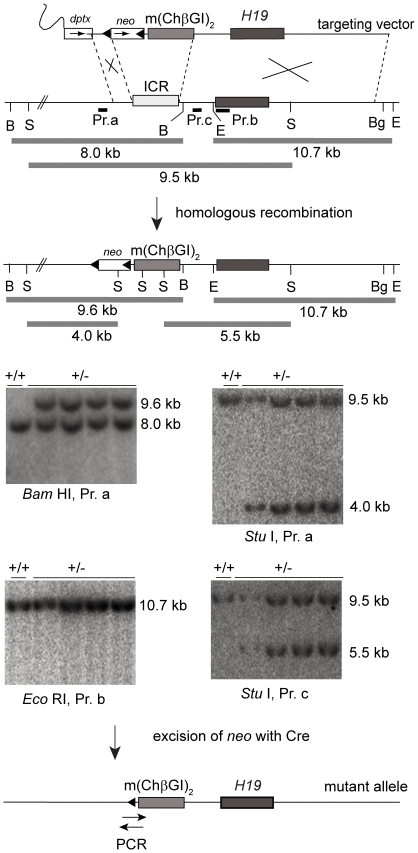
Targeting the (mChβGI)2 to the *H19/Igf2* ICR. The ICR was replaced by the (mChβGI)_2._ The CTCF sites were in the same orientation as the endogenous CTCF sites in the ICR. Novel restriction sites, such as *Stu*I were generated at the sites of the binding site deletions. One control +/+ and four +/− ES cell clones are shown out of 24 that underwent conservative recombination. The *lox*P sites-flanked *neo* selection cassette was removed by *Cre*-mediated excision.

The 2.2 kb long *Bam*HI fragment of (mChβGI)_2_ from pGEM4Z-Link2 was ligated into the *Bgl*II site of the *H19* ICR targeting vector [45]. The direction of the insert was verified by *Nhe*I digestion. Gene targeting was done in ES cells as before [39], [44], [45]. 96 neo positive ES cell clones were screened by PCR and verified by Southern blot hybridization (Figure 2). Probe a was a 0.5 kb PCR fragment made with primers 5′-GGTGCCATCAAGCTACTACAC-3′ and 5′-CTGGATAGGACATGGGCACAG-3′; probe b was a *Bam*HI-*Eco*RI restriction fragment and probe c was a *Sca*I-*Eco*RI restriction fragment. From 26 PCR-positive clones 24 clones underwent conservative recombination. Four ES cell lines were injected into 8-cell morulas and 2, 3, 1 and 9 chimeras were obtained from ES cell lines #1, #2, #22 and #29, respectively. None of the male chimeras produced viable mutant offspring. Male chimeras from independent ES clones did transmit the mutation, because we found fetuses positive for the mutation at 18.5 and 19.5 dpc in females pregnant from one chimera of ES#2 and three chimeras of ES#29 origin. One male chimera from ES#29 had a litter of 5 dead newborns, all positive for the mutation. Fetuses fathered by one other male chimera of ES#29 origin were systematically investigated (Table 1). Two chimeras from two independent ES cell lines (#22 and #29 origin) were female and produced male and female live F1. The *neo* cassette was removed by mating the female chimaeras with *Hprt-Cre* males of 129S1 genetic background [46]. Removal of the neo cassette was verified by the presence of a 0.24 kb PCR fragment spanning the remaining *lox*P site using primers 5′-GCCCACCAGCTGCTAGCCATC-3′ and 5′-CCTAGAGAATTCGAGGGACCTAATAAC-3′. Male F1 mutant did not produce live mutant offspring whereas female mutants transmitted the mutation. The *Hprt-Cre* gene was removed by mating of the F1 females with 129S1 males and was confirmed by PCR. The mutation from ES#29 was kept in the 129S1 strain in –(M)/+ form. Male mutants from this line were bred to females of different genetic background including 129S1, FVB inbred lines and CF1 outbred mice. Mutant pups never survived beyond day 1 after birth. Positive mice were identified by PCR ChβglU: 5′-TGTCTCAGTGTAAAGCCATTCC-3′ and ChβglL: 5′-TAACTTGCTCTTTGTCCTTCTATCC-3′.

**Table 1:** Weight of 18.5 dpc fetuses on maternal and paternal inheritance of the (mChβGI)_2._

	Mean wet weight (g) ± s.d. (n) (range) (% of +/+ weight)	
	fetus	placenta
+/+∧	1.313±0.098 (24) (1.166∼1.530)	0.066±0.009 (24) (0.053∼0.096)
-(M)/+∧	1.293±0.155 (24) (0.871∼1.511) (98.5%)	0.060±0.008 (24) (0.047∼0.077) (90.9%)
+/+‡	1.436±0.128 (13) (1.233∼1.593)	0.057±0.014 (13) (0.046∼0.101)
+/−(P)‡	0.635±0.055 (9) ¶ (0.571∼0.718) (44.2%)	0.032±0.007 (9) ¶ (0.024∼0.044) (56.1%)
+/+*	1.465±0.117 (19) (1.267∼1.692)	0.062±0.022 (19) (0.038∼0.109)
+/−(P)*	0.653±0.068 (16) ¶ (0.497∼0.755) (44.5%)	0.046±0.019 (16) $ (0.022∼0.083) (74.2%)
+/+†	1.126±0.194 (14) (0.873∼1.539)	0.077±0.011 (14) (0.057∼0.092)
+/−(P)†	0.564±0.071 (18) ¶ (0.486∼0.705) (50.1%)	0.038±0.006 (17) ¶ (0.030∼0.051) (49.4%)

∧ Sibling from -/(M) ♀ X +/+ ♂ matings.

‡ Sibling from +/+ ♀ X +/−(P) ♂ matings.

* Sibling from +/+ ♀ X +/−(P chimera #2) ♂ matings.

† Sibling from +/+ ♀ X +/−(P) ♂ matings. +/+ females were from transgenic line TgOG2.

¶ *P*<0.0001.

$ *P*<0.025.

(M), Maternal allele; (P), Paternal allele.

### Breeding of fetuses carrying the (mChβGI)_2_ for analysis

To produce the fetuses analyzed, one set of parents were males and females carrying the (mChβGI)_2_. These were F1N3-N4 descendants of a female chimera from ES clone #29. These were -(M)/+ heterozygous with respect to the (mChβGI)_2_, and lacked the *neo* cassette and the *Hprt-Cre* cassette and were in the 129S1 background. The other set of parents, unless stated otherwise were homozygous for the *Mus musculus castaneus* form of distal chromosome 7, as derived from CAST/Ei (CS). These were of strain FVB/NJ.CS(N7)-*distalChr.7^CS/CS^* [44]. The use of this cross allowed for allele-specific analysis of expression and DNA methylation. Hereafter, heterozygous fetuses maternally and paternally inheriting the (mChβGI)_2_ are designated -(M)/+ and +/−(P), respectively.

### Allele-specific gene expression by Sequenom SNuPE

Allele-specific *H19* and *Igf2* RNA gene expression analysis was based on SNPs between of inbred 129S1 (129) and CAST/Ei (CS) mouse strains and was analyzed by reverse-transcription PCR SNuPE assays [44], [47], except mass spectrometry quantified the extension primers (EP) based on molecular mass difference between alleles [48], [49]. Primers were designed using MassArray Assay v3.1. *H19:* 5′-ACGTTGGATGGCTTTGAGTCTCTCCGTATG-3′ 5′-ACGTTGGATGATGGACGACAGGTGGGTACT-3′and 5′-ATGTATACAGCGAGTGTG-3′ *Igf2*: 5′-ACGTTGGATGACATCAGGCTGTTCCCCTTG-3′ 5′-ACGTTGGATGGGGTTGTTTAGAGCCAATCA-3′and 5′-CCAATCAAATTTGGTTTTTTAGAA-3′. Amplified samples were spotted onto a 384 SpectroCHIP Array. Automated spectra acquisition was performed in a MassArray Compact mass spectrometer (Sequenom) using the Spectroacquire program (Sequenom) and was analyzed by MassArray Typer v3.4. We applied skew correction using a true heterozygote DNA sample to correct for any allelic imbalance in the SNP allele products. The % expression of each allele in the total expression was calculated at each given SNP.

### RNA Isolation and RT-PCR

RNA was isolated from using RNA-Bee (Tel-Test). Contaminating DNA was removed with the DNA-free Kit (Ambion). Reverse transcription was performed using equal amount of RNA using the Superscript III Random Primer Synthesis kit (Invitrogen). RT-PCR primers and probes were: *Igf2* exon 2–3: 5′-GGACCGCGGCTTCTACTTC-3′ 5′-AGCAGCACTCTTCCACGATG-3′, *Igf2* HEX: 5′-CCTTCAAGCCGTGCCAACCGTCGC-3′; this assay detects each possible alternative transcript because the primers are located in the common exons. *H19* exon 4–5: 5′-CTGAATCAAGAAGATGCTGCAATC-3′; 5′-GGTGCTATGAGTCTGCTCTTTC-3′; *H19* FAM: 5′-TGCCTCAGGAATCTGCTCCAAGGTG-3′; *Gapdh* exon 5–6:5′-AATGTGTCCGTCGTGGATCTG-3′; 5′-CAACCTGGTCCTCAGTGTAGC-3′; *Gapdh* Cy5: 5′-CGTGCCGCCTGGAGAAACCTGCC-3′.

### Purification of germ cells by flow cytometry

Male -(M)/+ heterozygous mice carrying the (mChβGI)_2_ were crossed with female homozygous transgenic mice of the TgOG2 line, which expresses the EGFP reporter gene specifically in germ cells [50]. From the resulting fetuses female or male germ cells were collected and purified by flow sorting as before [39], [44], [50].

### Methylation analysis by Southern hybridization

DNA was digested with *Bam*HI and *Bgl*II and with control *Msp*I (methylation non-sensitive) or *Hpa*II (methylation sensitive) enzymes. The mChβGI was labeled for hybridization probe. After *Hpa*II digestion, the probe visualized four bands: 1.45 kb (weak band due to short overlap with the probe), 800 bp, 700 bp and 350 bp, the same bands as after *Msp*I digestion. Therefore, the mChβGI was unmethylated.

### Bisulfite genomic sequencing

200 ng genomic DNA from fetal organs or 10,000–23,000 flow-sorted germ cells were used per bisulfite reaction performed in agarose beads as before [39] according to Olek et al [51]. Nested bisulfite primers for the (mChβGI)_2_ amplified the junction of the two insulators: U1: 5′-TTTTTTGGAGAAGGTAAATTTT-3′; L1: 5′-AATTAATAACCCTACACATAACAA-3′; U2: 5′- AAGGTTATTATTTTTTATTTAATTTTAG-3 and L2: 5′- ATAACAAAAAATTAAATCTAAATAAAC-3′.

## Results

### Replacing the *H19/Igf2* ICR with two copies of the mutant chicken β-globin insulator

We deleted the VEZF1 and USF1 binding sites from the 1.2 kb (ChβGI) using site-directed mutagenesis. The correctly mutagenized (mChβGI) was identified by restriction digestion and verified by DNA sequencing (Figure 1E). Two copies of this mutant insulator, (mChβGI)_2_ were introduced into mice to replace the *H19*/*Igf2* ICR by gene targeting (Figure 2). The (mChβGI)_2_ still harbored two functional CTCF binding sites and a high density of methylatable CpGs.

### Maternal inheritance of the (mChβGI)_2_

**Figure 3:**
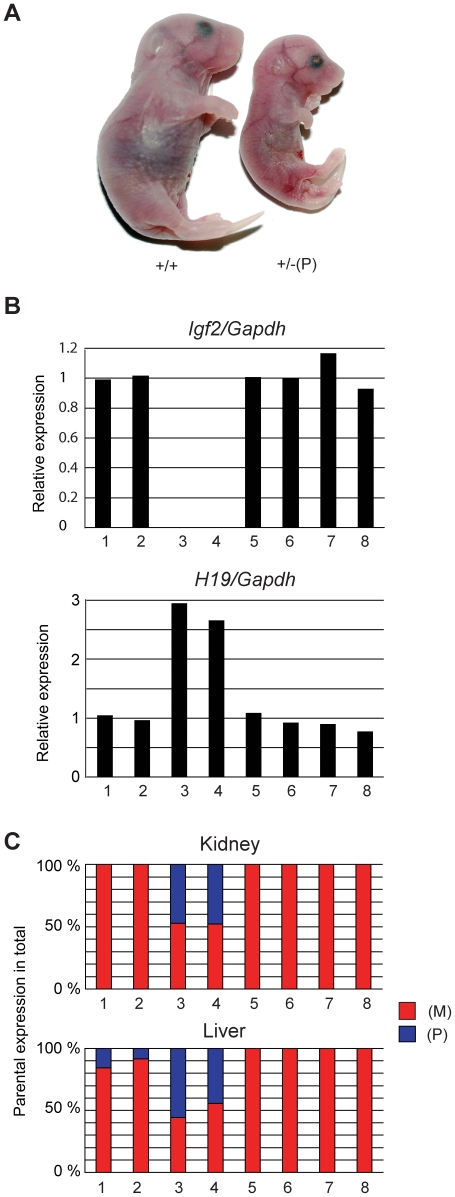
Phenotype of 18.5 dpc fetuses inheriting the (mChβGI)2. (A) Representative +/+ and +/–(P) fetuses are shown. (B) Expression of *Igf2* and *H19* was measured by real-time RT-PCR in kidneys. RNA from two +/–(P) and –(M)/+ fetuses (samples 3–4 and 7–8, respectively) and their +/+ littermates (samples 1–2 and 5–6) was analyzed. (C) Allele-specific expression of *H19* in kidneys and livers of the same fetuses was measured by RT-PCR SNuPE. The % expression of the maternal (M) and paternal (P) allele in the total expression is shown.

**Figure 4:**
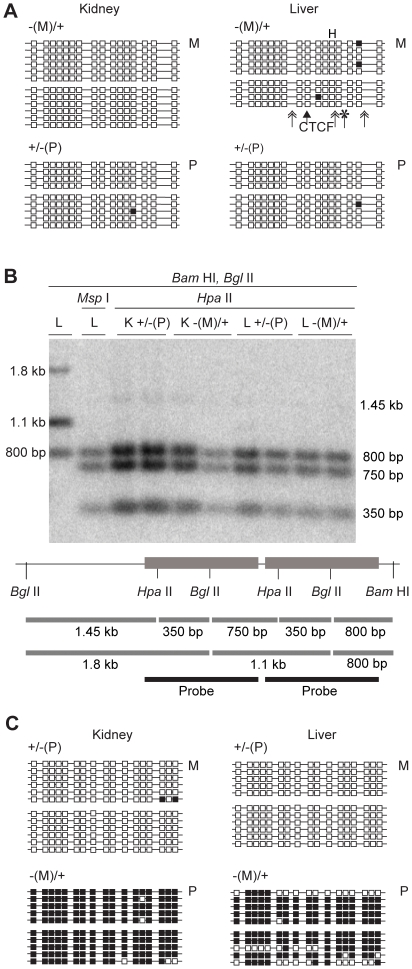
DNA methylation of the (mChβGI)2 in 18.5 dpc fetuses. (A) Bisulfite sequencing was performed to analyze CpG methylation of the (mChβGI)_2_ using genomic DNA from 18.5 dpc fetuses. Genotypes are indicated on top. Maternal (M) or paternal (P) transmission of the allele is indicated on the right. Unmethylated CpGs (white squares) and methylated CpGs (black squares) are shown along independent chromosomes (horizontal lines). Two siblings were assessed in each case, separated by space between groups of chromosomes. Simple arrow indicates the CTCF site. Double arrows and asterisk indicate the positions of the USF1 and VEZF1 deletions. (B) Southern blot hybridization results in kidneys (K) and livers (L) after paternal and maternal transmission. The (ChβGI) sequence was used as a probe. The two diagnostic *Hpa*II/*Msp*I sites and the *Bam*HI and *Bgl*II restriction sites are indicated. (C) Bisulfite sequencing of the ICR sequences from the same samples as in (A).

**Figure 5:**
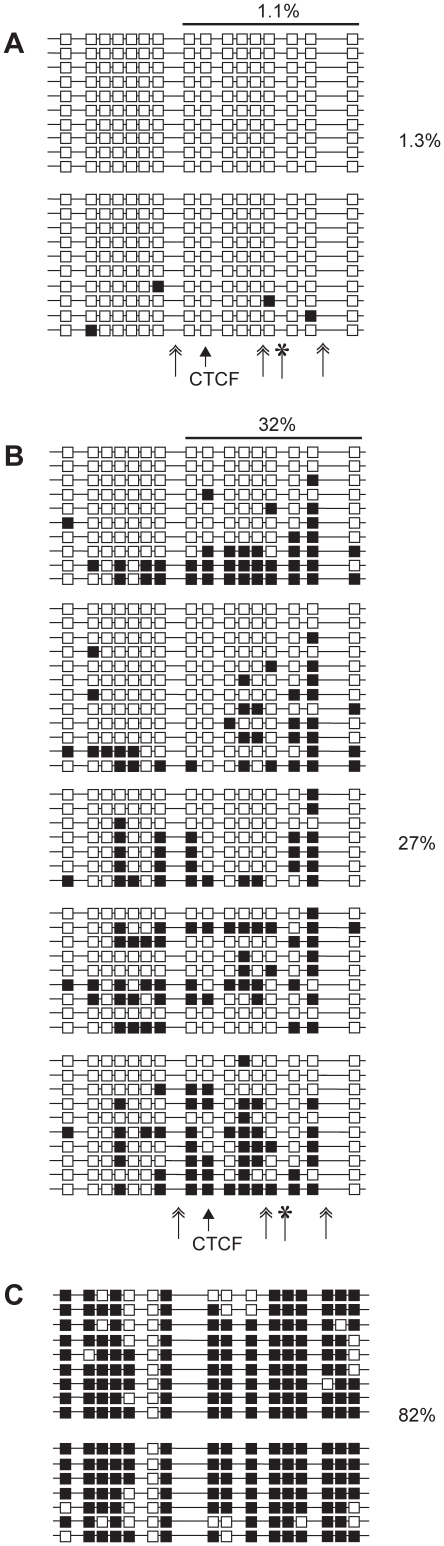
DNA methylation of the (mChβGI)2 in 18.5 dpc fetal germ cells. Bisulfite sequencing results are shown from +/−(P) fetuses. (A) The paternally inherited (mChβGI)_2_ allele in female germ cells. (B) The paternally inherited (mChβGI)_2_ allele in male germ cells. (C) The maternally inherited ICR sequences in male germ cells are shown as controls. The percentage of methylated CpGs is indicated for each allele. The bar above indicates the position of the previously analyzed CpGs [44] with the % of methylated CpGs in this subset. Chromosomes from independent bisulfite reactions are grouped. Other details are as in Figure 4.

Maternal inheritance resulted in normal and viable mice. The size of the –(M)/+ fetuses was normal (Table 1). The parental allele-specific expression patterns of *H19* and *Igf2* were normal. *H19* was expressed from the maternal allele in livers and kidneys of -(M)/+ 18.5 days post coitum (dpc) fetuses (Figure 3C) whereas *Igf2* was paternally expressed (data not shown) in the same samples. The (mChβGI)_2_, therefore perfectly substituted for the insulation function of the ICR, just as the (ChβGI)_2_ did. The (mChβGI)_2_ DNA, including the two CTCF binding sites, was unmethylated in somatic organs, kidneys and livers, of perinatal -(M)/+ fetuses (Figure 4A and B) similarly to the maternally inherited ICR (Figure 4C). The (mChβGI)_2_ correctly did not attain de novo methylation in the germ cells of female +/−(P) fetuses (Figure 5A). Therefore, the USF1 and VEZF1 binding sites were not required for protecting the (ChβGI)_2_ sequences from methylation in the fetal female germ line and in the maternal allele in the soma.

### Paternal inheritance of the (mChβGI)_2_

Out of 19 litters in different genetic backgrounds, we obtained 101 +/+ weanlings (Table 2). The expected number of +/− (P) weanlings was 101, but 0 was found. We inspected each cage on the day of birth. A total of 13 +/− (P) pups were found dead or died within a few hours after birth, consistent with a late fetal/perinatal lethality phenotype. Despite maternal attention, milk was not found in the stomach of the live newborns, indicating inability for feeding. Detailed histology was performed on coronal sections of the head and longitudinal sections of the body. Apart from the small size of body and organs there was no abnormality present in +/− (P) newborns (data not shown). 18.5 dpc +/−(P) fetuses were small (Table 1), but of normal appearance (Figure 3A). The weight of +/− (P) fetuses was 44% or 50% of the +/+ siblings depending on whether the mother was of CS or TgOG2 mouse strain. Placenta weight was also reduced (Table 1). Independently targeted ES cells gave similar results: +/− (P) fetuses from a male chimera (ES#2) or from male descendants of a female chimera (ES#29) were small (Table 1). The phenotype of +/(mChβGI)_2_ was more severe than that of +/(ChβGI)_2_ [44], where fetus weight at 18.5 dpc was 62% and 50–61% of +/+ littermates in the respective CS and TgOG2 crosses, and *Igf2* levels were 10%. *Igf2* expression was undetectable in +/(mChβGI)_2_ fetuses (Figure 3B) indicating that enhancer blocking by (mChβGI)_2_ was more complete in the absence of the USF1 and VEZF1 binding sites at the ICR position.

**Table 2:** Paternal transmission results in late fetal/neonatal lethality phenotype.

Mother	Offspring from m(ChβGI)_2_ fathers	
	+/+	+/−(P)
CF1	3	0 (1†)
	9	0
	8	0
	4	0
	10	0 (7††)
	7	0
	8	0
FVB	4	0
	7	0
	4	0 (3†)
	2	0
	4	0
	5	0
129S1	3	0
	4	0
	5	0 (2†)
	5	0
	5	0
	4	0
Total	101	0 (13^†^)

Normal (+/+) outbred CF1 and inbred, 129S1 and FVB, mothers were crossed with m(ChβGI)_2_/+ fathers and the offspring was genotyped at weaning. The number of wild type +/+ and mutant +/−(P) heterozygous young from each litter is given per row. Numbers in parentheses

† indicate dead pups of greatly reduced size, found on the day of birth.

Similarly to the (ChβGI)_2_ [44], the paternally inherited (mChβGI)_2_ was unmethylated in fetal organs (Figure 4A and B), indicating that the USF1 and VEZF sites were dispensable for hypomethylation of the (mChβGI)_2_ in the soma. The (mChβGI)_2_ DNA was more methylated than the (ChβGI)_2_ in male germ cells (32% versus 11% of CpGs methylated) (Figure 5B) [44], suggesting that the USF1 and/or VEZF1 proteins contributed to protecting the (ChβGI)_2_ sequence from de novo methylation in the male germ line. The (mChβGI)_2_, however, was less methylated than the normal ICR [52], [53] (Figure 5C), suggesting that it lacks the sequences that trigger full methylation of the ICR in prospermatogonia. The fact that partial methylation was attained in the male germ line (Figure 5B) but it was not observed in the soma (Figure 4A and B) demonstrates that the (ChβGI)_2_ DNA lacks the potential to maintain methylation in the paternal allele.

In the kidney and liver of +/− (P) perinatal fetuses, *H19* levels were more than 2-fold than in normal siblings (Figure 3B) and *H19* was biallelically expressed (Figure 3C) indicating that, unlike the fully methylated ICR, the hypomethylated (mChβGI)_2_ was not capable of inactivating the *H19* promoter in the paternal allele during post-fertilization development. Contrary to the (ChβGI)_2_, the (mChβGI)_2_ did not overactivate the *H19* in the paternal allele relative to the maternal allele. The paternal and maternal *H19* alleles each contributed 50% of total *H19* expression (Figure 3C). This suggests that USF and VEZF1 proteins in the (ChβGI)_2_ were responsible for overactivating *H19* in cis.

## Discussion

In this study we dissected the insulator and barrier functions of the (ChβGI) by deleting the USF and VEZF1 binding sites from the (ChβGI)_2_ and used two copies of this mutant chicken β-globin insulator (mChβGI)_2_ to substitute for the *H19*/*Igf2* ICR. Our results have implications for understanding insulator function and imprint establishment. The observed lethality phenotype argues that strict biallelic insulation at the ICR position is not tolerated in mouse development.

### On Insulator Function

Insulators are often complex, harboring enhancer blocking and chromatin barrier activities. We genetically dissected the (ChβGI)_2_, and tested whether its barrier function is required for substituting the *H19/Igf2* ICR. The (mChβGI)_2_ insulated the *Igf2* promoter from the shared enhancers, suggesting that CTCF binding is sufficient and the VEZF1 and USF barrier proteins are dispensable for insulation at the *H19*/*Igf2* ICR position. Whereas 10% *Igf2* residual expression remained in livers and kidneys of +/(ChβGI)_2_ fetuses and 75 and 95% of this was from the paternal allele, respectively (Szabó, PE and Mann, JR, unpublished) indicating incomplete insulation, *Igf2* RNA was undetectable in +/(mChβGI)_2_ fetuses, indicating complete insulation. A stronger enhancer blocker function was, therefore, achieved by CTCF alone than by using a combination of CTCF, USF and VEZF sites.

Barrier proteins, USF and VEZF1, do not insulate enhancers from promoters but protect surrounded transgenes from the invasion of heterochromatin: they maintain active chromatin by recruiting histone acetyltransferases (HATs) and also protect the DNA from de novo methylation. USF directly recruits HATs p300/CBP and PCAF and H3K4 methyltransferase Set7/9 to enforce active chromatin [22], [42]. VEZF1 is important for maintaining euchromatin [22] and DNA hypomethylation at the ChβGI and along the ChβGI-surrounded transgene [21]. CTCF protein, apart from its enhancer blocking function, has very similar activities. CTCF maintains ICR hypomethylation in somatic cells [34], [35], [37], [38], [39]. CTCF can recruit the HAT, CHD8 to the ICR [54]. In the maternal allele CTCF recruits active histone tail modification marks to the ICR and to the *H19* gene [31] and also recruits at a distance, Polycomb-mediated H3K27me3 repressive marks at the *Igf2* promoter and at the *Igf2* DMRs [31], [33]. Further studies will be required to fully understand the molecular mechanisms of chromatin barrier versus enhancer blocking functions. It will be interesting to see if chromatin barriers exist in vertebrates without CTCF. It will be interesting for example, to compare the in vivo occupancy of CTCF binding sites with VEZF1 and USF1 sites in a genome-wide study. CTCF alone may insulate by enhancer blocking but in combination with barrier proteins it may insulate by forming chromatin barriers. Because VEZF1 and USF barrier proteins were dispensable for insulation at the ICR position, chromatin barrier formation in the maternal allele may not be required at all for proper regulation of imprinted genes at the ICR position.

We find it interesting that whereas CTCF is required for protecting the ICR from methylation in the soma [35], [38], [39], it doesn't protect from methylation imprint establishment the male germ line [35], [39]. USF and VEZF1 sites, on the other hand, are not required for protecting the (ChβGI)_2_ from methylation in the soma but contribute to its protection in the male germ line at the ICR position. In fetal male germ cells CTCF protein may not bind in the ICR whereas USF and/or VEZF1 proteins may bind in the (ChβGI)_2_. Chromatin analysis in fetal germ cells will be needed to follow up on these possibilities.

### On Imprint Establishment

The mechanism that targets DNA methylation imprint establishment to the *H19/Igf2* ICR in fetal male germ cells is still unknown. Tandem repeats in this domain have no role in methylation targeting [55], [56], [57]. Mutagenesis of specific protein binding sites had no effect on DNA methylation imprint establishment at the ICR: methylation was undisturbed in mutant male germ cells and lack of methylation was undisturbed in mutant female germ cells [45], [58], [59], [60]. Whereas the ICR became methylated in male germ cells in a randomly integrated 150 kb *H19* transgene, it did not attain DNA methylation or accumulated only partial methylation in the male germ line when introduced to genomic locations other than at the *H19* locus [61], [62], [63], [64]. These results suggest that the genomic location is important for methylation imprint establishment of the ICR in the male germ line.

Our study provides indirect clues to the question whether the genomic locus or the *H19/Igf2* ICR sequence determines DNA methylation imprint establishment in the germ line. We found that the (mChβGI)_2_ became partially (32%) methylated in male germ cells but remained unmethylated in female germ cells. This male germ cell-specific methylation of the (mChβGI)_2_ is consistent with the possibility that the genomic locus carries “methylator elements” that target Dnmt3a and Dnmt3L to the ICR position. Parental specific methylation can occur on integrated transgenes [65], [66], [67], [68]. The genomic location could target de novo methylation to the ICR for example by an RNA that is transcribed across the ICR specifically in male fetal germ cells, similarly to the *Gnas* DMRs in growing oocytes [69]. The (mChβGI)_2_ attained 3-fold higher CpG methylation in prospermatogonia than the (ChβGI)_2_, suggesting that the boundary proteins USF1 and VEZF1 provided the (ChβGI)_2_ protection from de novo methylation in the male germ line. Unlike the endogenous ICR sequence, however, the mutant (mChβGI)_2_ was not fully methylated, indicating that the (mChβGI)_2_ may be missing sequence elements that target methylation to the ICR in prospermatogonia.

Whereas the (mChβGI)_2_ attained 32% de novo male germ cell-specific mehylation, it was unmethylated in the paternally inherited allele in fetal somatic organs. The (mChβGI)_2_, therefore, lacks the potential of methylation imprint maintenance at the *H19*/*Igf2* locus. CpG methylation is likely lost during the global wave of epigenetic remodeling events in the embryo. A methylation maintaining role of 9 CpGs in the 4 CTCF sites has been confirmed in the ICR [36], but 2 CpG-s in the (mChβGI)_2_ CTCF sites did not fulfill this role. Alternatively, the level of methylation has to be over a threshold at the ICR position to be recognized for maintenance.

### On the Lethality Phenotype

Paternal inheritance of (mChβGI)_2_ resulted in a more severe phenotype than that of (ChβGI)_2_, causing not only smaller fetus size but also perinatal death. The lethality phenotype cannot be explained by the absence of the paternally inherited ICR, because paternal deletion of the ICR [28] or its substitution with the (ChβGI)_2_ [44] does not cause lethality.

The insulator function of the (ChβGI)_2_ became even stronger in the absence of the barrier proteins as illustrated by the levels of *Igf2* expression. IGF2 is an embryonic and fetal mitogen [70], [71] and is also important for placenta development [72]. Therefore, the difference in *Igf2* expression (10% versus 0%) likely accounts for the weight difference between the +/(ChβGI)_2_ and the +/(mChβGI)_2_ fetuses (50–61% versus 44–50%) and placentas (60% versus 56%), respectively. The lethality phenotype, however, cannot be explained by lack of *Igf2*, because although *Igf2* +/−(P) and *Igf2* −/− mice are small (50–62%), they are viable [70], [71], [73]. Our data argue that biallelic strict insulation at the ICR position is the cause of lethality in +/(mChβGI)_2_ pups by causing misexpression of at least one gene in addition to *Igf2*.

Allele-specific insulation by the ICR likely affects a number of transcripts in the imprinted domain apart from *H19* and *Igf2* in the fetus and *Insulin 2* (*Ins2*) in the placenta [74]. These transcripts, *H19* microRNA (*Mir675*) [75], *Igf2* antisense RNAs (*Igf2as*) [76], [77] and *Mir483* within an intron of *Igf2* [78] could be also misregulated by biallelic insulation. Bi-maternal misexpression of one or more of these transcripts (too much *H19* or *Mir675* or missing *Igf2as*, *Mir483* or *Ins2*) or other, yet unidentied ICR-controlled transcripts, by strict biallelic insulation must contribute to the death of +/(mChβGI)_2_ pups. The *H19* noncoding RNA has been suggested to regulate an imprinted gene network [79].

**Figure 6:**
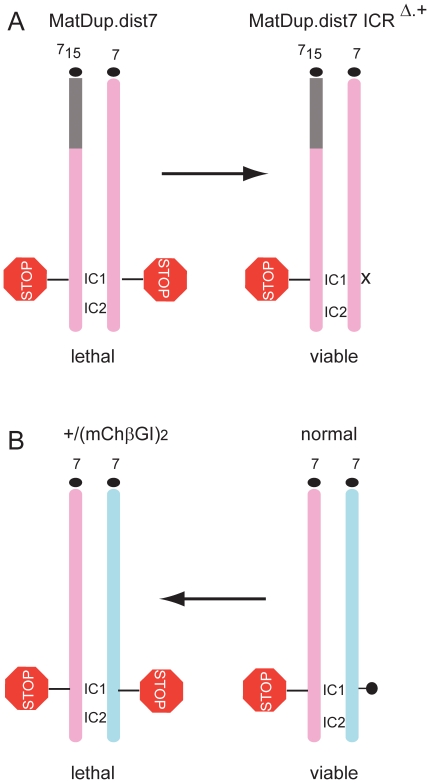
Lethality is caused by strict biallelic insulation at the IC1 in mouse chromosome 7. (A) Maternal (pink) duplication of distal chromosome 7 (MatDup.dist7) fetuses carry biallelic insulation (STOP signal) at the imprinting control center 1 (IC1) and die. The lethality phenotype is rescued by maternal transmission of one copy of the mutant IC1 that lacks CTCF binding (x) and insulator function [81]. The imprinting control center 2 (IC2) is bi-maternal. Correction of biallelic ICR insulation to monoallelic insulation is sufficient to rescue perinatal lethality of the MatDup.dist7 genotype. (B) Our present experiments provide the reciprocal argument: introducing strict biallelic insulation to the IC1 causes lethality. By substituting the paternal chromosome's (light blue) methylated (black lollipop) ICR with the unmethylated (ChβGI)_2_ [44] or the (mChβGI)_2_ we introduced biallelic insulation into the IC1 and IC2 remained intact. Lethality was observed in the +/(mChβGI)_2_ but not in the +/(ChβGI)_2_ genotype 44]. By removing the USF and VEZF1 binding sites from the (ChβGI)_2_, biallelic insulation has become complete, causing death in the +/(mChβGI)_2_ genotype.

Maternal duplication of chromosome 7 distal to the T9H translocation breakpoint (MatDup.dist7) [80], [81], [82] exhibits small fetus weight (about 40%), undetectable *Igf2* expression and late fetal/perinatal lethality [81], [83]. In MatDup.dist7 fetuses, bi-maternal misexpression of imprinted genes occur within the influence of the ICR [81], also called imprinting control center 1 (IC1) and outside the influence of the ICR for example under the control of the KvDMR1 or imprinting control center 2 (IC2) [84], [85]. Yet, none of the tested bi-maternal misexpressions causes death [81]. The lethality phenotype of MatDup.dist7 was completely rescued by maternal transmission of the mutant *H19/Igf2* ICR that lacks CTCF binding and, therefore, lacks insulator function [81], suggesting that correction of biallelic ICR insulation to monoallelic insulation at the IC1 is sufficient to rescue the perinatal lethality of the MatDup.dist7 genotype (Figure 6A). The reciprocal experiment, introducing biallelic insulation at the IC1, did not, at first, cause death [44], suggesting then that biallelic insulation by IC1 and additional misexpressions in distal chromosome 7 are responsible for the MatDup.dist7 lethality. In the present study, by substituting the paternal ICR with the (mChβGI)_2_ lacking the USF and VEZF1 binding sites, a complete biallelic insulation was achieved at the IC1 and this resulted in lethality in the +/(mChβGI)_2_ genotype (Figure 6B). Our present experiment, therefore, is consistent with the explanation that the lethality of the MatDup.dist7 genotype is caused by misregulation of *Igf2* and something else under the control of the IC1 and is not dependent on genes outside of the control of IC1. Similarly, bi-maternal insulation by the IC1 can explain the perinatal lethality of bi-maternal ngΔ12/fg fetuses produced from a non-growing oocyte genome carrying an IG-DMR deletion in chromosome 12 and a fully grown oocyte genome [86].

The present mouse mutation will be a useful animal model for understanding the severe form of Silver-Russell syndrome (SRS) (OMIM 180860) [87]. SRS is characterized by intrauterine and postnatal growth retardation and in the majority of cases is associated with hypomethylation of the ICR. The severity of low birth weight phenotype in SRS correlates with the level of ICR hypomethylation [88] and likely correlates with insulator strength, because CTCF binding in the ICR is methylation sensitive [23], [24], [25], [26]. In our mouse models, the decision between life and death depended on insulator strength. The barrier proteins, VEZF1 and USF can rescue lethality by reducing the insulator strength at the IC1 position by 10%. It is not known if small fetus/placenta weight *per se* causes stillbirth in humans, but intrauterine growth restriction/placental insufficiency was diagnosed in 23% of human stillbirth cases in a recent study [89]. We predict that the most severe cases of SRS—which would be expected to have a complete lack of methylation at the ICR and strict biallelic insulation— do not survive to term or die around birth.

## References

[1] Dorman ER, Bushey AM, Corces VG (2007). The role of insulator elements in large-scale chromatin structure in interphase.. Semin Cell Dev Biol.

[2] Felsenfeld G, Burgess-Beusse B, Farrell C, Gaszner M, Ghirlando R (2004). Chromatin boundaries and chromatin domains.. Cold Spring Harb Symp Quant Biol.

[3] Gerasimova TI, Corces VG (1996). Boundary and insulator elements in chromosomes.. Curr Opin Genet Dev.

[4] West AG, Fraser P (2005). Remote control of gene transcription.. Hum Mol Genet 14 Spec No.

[5] Bell AC, West AG, Felsenfeld G (1999). The protein CTCF is required for the enhancer blocking activity of vertebrate insulators.. Cell.

[6] Moon H, Filippova G, Loukinov D, Pugacheva E, Chen Q (2005). CTCF is conserved from Drosophila to humans and confers enhancer blocking of the Fab-8 insulator.. EMBO Rep.

[7] Filippova GN, Fagerlie S, Klenova EM, Myers C, Dehner Y (1996). An exceptionally conserved transcriptional repressor, CTCF, employs different combinations of zinc fingers to bind diverged promoter sequences of avian and mammalian c-myc oncogenes.. Mol Cell Biol.

[8] Chung JH, Bell AC, Felsenfeld G (1997). Characterization of the chicken beta-globin insulator.. Proc Natl Acad Sci U S A.

[9] Chung JH, Whiteley M, Felsenfeld G (1993). A 5′ element of the chicken beta-globin domain serves as an insulator in human erythroid cells and protects against position effect in Drosophila.. Cell.

[10] Barski A, Cuddapah S, Cui K, Roh TY, Schones DE (2007). High-resolution profiling of histone methylations in the human genome.. Cell.

[11] Cuddapah S, Jothi R, Schones DE, Roh TY, Cui K (2009). Global analysis of the insulator binding protein CTCF in chromatin barrier regions reveals demarcation of active and repressive domains.. Genome Res.

[12] Recillas-Targa F, Pikaart MJ, Burgess-Beusse B, Bell AC, Litt MD (2002). Position-effect protection and enhancer blocking by the chicken beta-globin insulator are separable activities.. Proc Natl Acad Sci U S A.

[13] Sawadogo M, Roeder RG (1985). Interaction of a gene-specific transcription factor with the adenovirus major late promoter upstream of the TATA box region.. Cell.

[14] Aitsebaomo J, Kingsley-Kallesen ML, Wu Y, Quertermous T, Patterson C (2001). Vezf1/DB1 is an endothelial cell-specific transcription factor that regulates expression of the endothelin-1 promoter.. J Biol Chem.

[15] Koyano-Nakagawa N, Nishida J, Baldwin D, Arai K, Yokota T (1994). Molecular cloning of a novel human cDNA encoding a zinc finger protein that binds to the interleukin-3 promoter.. Mol Cell Biol.

[16] Kuhnert F, Campagnolo L, Xiong JW, Lemons D, Fitch MJ (2005). Dosage-dependent requirement for mouse Vezf1 in vascular system development.. Dev Biol.

[17] Xiong JW, Leahy A, Lee HH, Stuhlmann H (1999). Vezf1: A Zn finger transcription factor restricted to endothelial cells and their precursors.. Dev Biol.

[18] Clark SP, Lewis CD, Felsenfeld G (1990). Properties of BGP1, a poly(dG)-binding protein from chicken erythrocytes.. Nucleic Acids Res.

[19] Lewis CD, Clark SP, Felsenfeld G, Gould H (1988). An erythrocyte-specific protein that binds to the poly(dG) region of the chicken beta-globin gene promoter.. Genes Dev.

[20] Gowher H, Stuhlmann H, Felsenfeld G (2008). Vezf1 regulates genomic DNA methylation through its effects on expression of DNA methyltransferase Dnmt3b.. Genes Dev.

[21] Dickson J, Gowher H, Strogantsev R, Gaszner M, Hair A (2010). VEZF1 elements mediate protection from DNA methylation.. PLoS Genet.

[22] West AG, Huang S, Gaszner M, Litt MD, Felsenfeld G (2004). Recruitment of histone modifications by USF proteins at a vertebrate barrier element.. Mol Cell.

[23] Bell AC, Felsenfeld G (2000). Methylation of a CTCF-dependent boundary controls imprinted expression of the Igf2 gene.. Nature.

[24] Hark AT, Schoenherr CJ, Katz DJ, Ingram RS, Levorse JM (2000). CTCF mediates methylation-sensitive enhancer-blocking activity at the H19/Igf2 locus.. Nature.

[25] Kanduri C, Pant V, Loukinov D, Pugacheva E, Qi CF (2000). Functional association of CTCF with the insulator upstream of the H19 gene is parent of origin-specific and methylation-sensitive.. Curr Biol.

[26] Szabó P, Tang SH, Rentsendorj A, Pfeifer GP, Mann JR (2000). Maternal-specific footprints at putative CTCF sites in the H19 imprinting control region give evidence for insulator function.. Curr Biol.

[27] Bartolomei MS, Webber AL, Brunkow ME, Tilghman SM (1993). Epigenetic mechanisms underlying the imprinting of the mouse H19 gene.. Genes Dev.

[28] Thorvaldsen JL, Duran KL, Bartolomei MS (1998). Deletion of the H19 differentially methylated domain results in loss of imprinted expression of H19 and Igf2.. Genes Dev.

[29] Tremblay KD, Duran KL, Bartolomei MS (1997). A 5′ 2-kilobase-pair region of the imprinted mouse H19 gene exhibits exclusive paternal methylation throughout development.. Mol Cell Biol.

[30] Tremblay KD, Saam JR, Ingram RS, Tilghman SM, Bartolomei MS (1995). A paternal-specific methylation imprint marks the alleles of the mouse H19 gene.. Nat Genet.

[31] Han L, Lee DH, Szabó PE (2008). CTCF is the master organizer of domain-wide allele-specific chromatin at the H19/Igf2 imprinted region.. Mol Cell Biol.

[32] Kanduri M, Kanduri C, Mariano P, Vostrov AA, Quitschke W (2002). Multiple nucleosome positioning sites regulate the CTCF-mediated insulator function of the H19 imprinting control region.. Mol Cell Biol.

[33] Li T, Hu JF, Qiu X, Ling J, Chen H (2008). CTCF regulates allelic expression of Igf2 by orchestrating a promoter-polycomb repressive complex 2 intrachromosomal loop.. Mol Cell Biol.

[34] Pant V, Mariano P, Kanduri C, Mattsson A, Lobanenkov V (2003). The nucleotides responsible for the direct physical contact between the chromatin insulator protein CTCF and the H19 imprinting control region manifest parent of origin-specific long-distance insulation and methylation-free domains.. Genes Dev.

[35] Engel N, Thorvaldsen JL, Bartolomei MS (2006). CTCF binding sites promote transcription initiation and prevent DNA methylation on the maternal allele at the imprinted H19/Igf2 locus.. Hum Mol Genet.

[36] Engel N, West AG, Felsenfeld G, Bartolomei MS (2004). Antagonism between DNA hypermethylation and enhancer-blocking activity at the H19 DMD is uncovered by CpG mutations.. Nat Genet.

[37] Pant V, Kurukuti S, Pugacheva E, Shamsuddin S, Mariano P (2004). Mutation of a single CTCF target site within the H19 imprinting control region leads to loss of Igf2 imprinting and complex patterns of de novo methylation upon maternal inheritance.. Mol Cell Biol.

[38] Schoenherr CJ, Levorse JM, Tilghman SM (2003). CTCF maintains differential methylation at the Igf2/H19 locus.. Nat Genet.

[39] Szabó PE, Tang SH, Silva FJ, Tsark WM, Mann JR (2004). Role of CTCF binding sites in the Igf2/H19 imprinting control region.. Mol Cell Biol.

[40] Leighton PA, Saam JR, Ingram RS, Stewart CL, Tilghman SM (1995). An enhancer deletion affects both H19 and Igf2 expression.. Genes Dev.

[41] Srivastava M, Hsieh S, Grinberg A, Williams-Simons L, Huang SP (2000). H19 and Igf2 monoallelic expression is regulated in two distinct ways by a shared cis acting regulatory region upstream of H19.. Genes Dev.

[42] Mutskov VJ, Farrell CM, Wade PA, Wolffe AP, Felsenfeld G (2002). The barrier function of an insulator couples high histone acetylation levels with specific protection of promoter DNA from methylation.. Genes Dev.

[43] Pikaart MJ, Recillas-Targa F, Felsenfeld G (1998). Loss of transcriptional activity of a transgene is accompanied by DNA methylation and histone deacetylation and is prevented by insulators.. Genes Dev.

[44] Szabó PE, Tang SH, Reed MR, Silva FJ, Tsark WM (2002). The chicken beta-globin insulator element conveys chromatin boundary activity but not imprinting at the mouse Igf2/H19 domain.. Development.

[45] Szabó PE, Han L, Hyo-Jung J, Mann JR (2006). Mutagenesis in mice of nuclear hormone receptor binding sites in the Igf2/H19 imprinting control region.. Cytogenet Genome Res.

[46] Tang SH, Silva FJ, Tsark WM, Mann JR (2002). A Cre/loxP-deleter transgenic line in mouse strain 129S1/SvImJ.. Genesis.

[47] Szabó PE, Mann JR (1995). Biallelic expression of imprinted genes in the mouse germ line: implications for erasure, establishment, and mechanisms of genomic imprinting.. Genes Dev.

[48] Haun WJ, Springer NM (2008). Maternal and paternal alleles exhibit differential histone methylation and acetylation at maize imprinted genes.. Plant J.

[49] Jurinke C, Denissenko MF, Oeth P, Ehrich M, van den Boom D (2005). A single nucleotide polymorphism based approach for the identification and characterization of gene expression modulation using MassARRAY.. Mutat Res.

[50] Szabó PE, Hubner K, Scholer H, Mann JR (2002). Allele-specific expression of imprinted genes in mouse migratory primordial germ cells.. Mech Dev.

[51] Olek A, Oswald J, Walter J (1996). A modified and improved method for bisulphite based cytosine methylation analysis.. Nucleic Acids Res.

[52] Davis TL, Yang GJ, McCarrey JR, Bartolomei MS (2000). The H19 methylation imprint is erased and re-established differentially on the parental alleles during male germ cell development.. Hum Mol Genet.

[53] Ueda T, Abe K, Miura A, Yuzuriha M, Zubair M (2000). The paternal methylation imprint of the mouse H19 locus is acquired in the gonocyte stage during foetal testis development.. Genes Cells.

[54] Ishihara K, Oshimura M, Nakao M (2006). CTCF-dependent chromatin insulator is linked to epigenetic remodeling.. Mol Cell.

[55] Lewis A, Mitsuya K, Constancia M, Reik W (2004). Tandem repeat hypothesis in imprinting: deletion of a conserved direct repeat element upstream of H19 has no effect on imprinting in the Igf2-H19 region.. Mol Cell Biol.

[56] Reed MR, Riggs AD, Mann JR (2001). Deletion of a direct repeat element has no effect on Igf2 and H19 imprinting.. Mamm Genome.

[57] Stadnick MP, Pieracci FM, Cranston MJ, Taksel E, Thorvaldsen JL (1999). Role of a 461-bp G-rich repetitive element in H19 transgene imprinting.. Dev Genes Evol.

[58] Bowman AB, Levorse JM, Ingram RS, Tilghman SM (2003). Functional characterization of a testis-specific DNA binding activity at the H19/Igf2 imprinting control region.. Mol Cell Biol.

[59] Katz DJ, Beer MA, Levorse JM, Tilghman SM (2005). Functional characterization of a novel Ku70/80 pause site at the H19/Igf2 imprinting control region.. Mol Cell Biol.

[60] Szabó PE, Pfeifer GP, Mann JR (2004). Parent-of-origin-specific binding of nuclear hormone receptor complexes in the H19-Igf2 imprinting control region.. Mol Cell Biol.

[61] Gebert C, Kunkel D, Grinberg A, Pfeifer K (2010). H19 imprinting control region methylation requires an imprinted environment only in the male germ line.. Mol Cell Biol.

[62] Park KY, Sellars EA, Grinberg A, Huang SP, Pfeifer K (2004). The H19 differentially methylated region marks the parental origin of a heterologous locus without gametic DNA methylation.. Mol Cell Biol.

[63] Reinhart B, Eljanne M, Chaillet JR (2002). Shared role for differentially methylated domains of imprinted genes.. Mol Cell Biol.

[64] Tanimoto K, Shimotsuma M, Matsuzaki H, Omori A, Bungert J (2005). Genomic imprinting recapitulated in the human {beta}-globin locus.. Proc Natl Acad Sci U S A.

[65] Reik W, Collick A, Norris ML, Barton SC, Surani MA (1987). Genomic imprinting determines methylation of parental alleles in transgenic mice.. Nature.

[66] Sapienza C, Peterson AC, Rossant J, Balling R (1987). Degree of methylation of transgenes is dependent on gamete of origin.. Nature.

[67] Swain JL, Stewart TA, Leder P (1987). Parental legacy determines methylation and expression of an autosomal transgene: a molecular mechanism for parental imprinting.. Cell.

[68] Ueda T, Yamazaki K, Suzuki R, Fujimoto H, Sasaki H (1992). Parental methylation patterns of a transgenic locus in adult somatic tissues are imprinted during gametogenesis.. Development.

[69] Chotalia M, Smallwood SA, Ruf N, Dawson C, Lucifero D (2009). Transcription is required for establishment of germline methylation marks at imprinted genes.. Genes Dev.

[70] DeChiara TM, Efstratiadis A, Robertson EJ (1990). A growth-deficiency phenotype in heterozygous mice carrying an insulin-like growth factor II gene disrupted by targeting.. Nature.

[71] DeChiara TM, Robertson EJ, Efstratiadis A (1991). Parental imprinting of the mouse insulin-like growth factor II gene.. Cell.

[72] Dilworth MR, Kusinski LC, Cowley E, Ward BS, Husain SM (2010). Placental-specific Igf2 knockout mice exhibit hypocalcemia and adaptive changes in placental calcium transport.. Proc Natl Acad Sci U S A.

[73] Murrell A, Heeson S, Bowden L, Constancia M, Dean W (2001). An intragenic methylated region in the imprinted Igf2 gene augments transcription.. EMBO Rep.

[74] Moore GE, Abu-Amero SN, Bell G, Wakeling EL, Kingsnorth A (2001). Evidence that insulin is imprinted in the human yolk sac.. Diabetes.

[75] Cai X, Cullen BR (2007). The imprinted H19 noncoding RNA is a primary microRNA precursor.. Rna.

[76] Moore T, Constancia M, Zubair M, Bailleul B, Feil R (1997). Multiple imprinted sense and antisense transcripts, differential methylation and tandem repeats in a putative imprinting control region upstream of mouse Igf2.. Proc Natl Acad Sci U S A.

[77] Monk D, Sanches R, Arnaud P, Apostolidou S, Hills FA (2006). Imprinting of IGF2 P0 transcript and novel alternatively spliced INS-IGF2 isoforms show differences between mouse and human.. Hum Mol Genet.

[78] Landgraf P, Rusu M, Sheridan R, Sewer A, Iovino N (2007). A mammalian microRNA expression atlas based on small RNA library sequencing.. Cell.

[79] Gabory A, Ripoche MA, Le Digarcher A, Watrin F, Ziyyat A (2009). H19 acts as a trans regulator of the imprinted gene network controlling growth in mice.. Development.

[80] Searle AG, Beechey CV (1990). Genome imprinting phenomena on mouse chromosome 7.. Genet Res.

[81] Han L, Szabó PE, Mann JR (2010). Postnatal survival of mice with maternal duplication of distal chromosome 7 induced by a Igf2/H19 imprinting control region lacking insulator function.. PLoS Genet.

[82] Ferguson-Smith AC, Cattanach BM, Barton SC, Beechey CV, Surani MA (1991). Embryological and molecular investigations of parental imprinting on mouse chromosome 7.. Nature.

[83] Beechey CV, Ball ST, Townsend KM, Jones J (1997). The mouse chromosome 7 distal imprinting domain maps to G-bands F4/F5.. Mamm Genome.

[84] Fitzpatrick GV, Soloway PD, Higgins MJ (2002). Regional loss of imprinting and growth deficiency in mice with a targeted deletion of KvDMR1.. Nat Genet.

[85] Mancini-Dinardo D, Steele SJ, Levorse JM, Ingram RS, Tilghman SM (2006). Elongation of the Kcnq1ot1 transcript is required for genomic imprinting of neighboring genes.. Genes Dev.

[86] Kawahara M, Wu Q, Ferguson-Smith AC, Kono T (2007). Appropriate expression of imprinted genes on mouse chromosome 12 extends development of bi-maternal embryos to term.. FEBS Lett.

[87] Eggermann T, Eggermann K, Schonherr N (2008). Growth retardation versus overgrowth: Silver-Russell syndrome is genetically opposite to Beckwith-Wiedemann syndrome.. Trends Genet.

[88] Bruce S, Hannula-Jouppi K, Peltonen J, Kere J, Lipsanen-Nyman M (2009). Clinically distinct epigenetic subgroups in Silver-Russell syndrome: the degree of H19 hypomethylation associates with phenotype severity and genital and skeletal anomalies.. J Clin Endocrinol Metab.

[89] Varli IH, Petersson K, Bottinga R, Bremme K, Hofsjo A (2008). The Stockholm classification of stillbirth.. Acta Obstet Gynecol Scand.

